# A Review of the Relationship of the Cerebrospinal Fluid Changes During the Dysregulation of Parathyroid Hormone With Psychiatric or Neurological Manifestations

**DOI:** 10.7759/cureus.12679

**Published:** 2021-01-13

**Authors:** Ifrah Kaleem, Josh Alexander, Mohamed Hisbulla, Vishmita Kannichamy, Vinayak Mishra, Amit Banerjee, Arohi B Gandhi, Safeera Khan

**Affiliations:** 1 Internal Medicine, California Institute of Behavioral Neurosciences & Psychology, Fairfield, USA; 2 Internal medicine, California Institute of Behavioral Neurosciences & Psychology, Fairfield, USA; 3 General Medicine, California Institute of Behavioral Neurosciences & Psychology, Fairfield, USA

**Keywords:** csf & pth, csf & psychosis, pth & psychosis, vitamin d & depression, calcium & schizophrenia, parathyroid hormone/cerebrospinal fluid"[mesh], cerebrospinal fluid/physiology"[mesh], psychiatry/physiology"[mesh], calcium/cerebrospinal fluid"[mesh]

## Abstract

It is established that normal calcium and vitamin D concentrations are maintained in the body through parathyroid hormone (PTH), a signaling molecule secreted from parathyroid glands. A massive role is played by PTH in increasing calcium levels when they are extremely low in the body through different mechanisms. The dysregulation of this hormone is due to either over functioning of the gland (hyperparathyroidism) or compromised functioning in hypoparathyroidism. A detailed review was done to identify if any changes are happening in the cerebrospinal fluid (CSF) due to any pathology causing the parathormone to be dysregulated enough to, in turn, cause any further pathology in the nervous system. This may then lead to various disabling neuropsychiatric features. The calcium and vitamin D abnormalities are both directly and indirectly connected to psychiatric features like delusions, schizophrenia, disabled cognition, psychosis, coma, mania, and depression of all kinds.

Moreover, their irregularities are also linked to Alzheimer's. During these manifestations, the CSF is altered concentration-wise, where elevated calcium levels inside are observed during different studies. Despite PTH's indirect connection to the CSF modifications, their association hasn't been potently proven yet, considering more observational studies should be conducted in humans and for a more extended period, along with bigger and greater numbers of CSF samples. Suppose there is a possibility of the link of CSF alterations to PTH. In that case, we can consider a pronounced increase of CSF calcium or PTH as a risk factor for debilitating neuropsychiatric diseases. In this review, the possible correlation of CSF and PTH has been discussed.

## Introduction and background

If you skew the endocrine system, you lose the pathways to self; when the endocrine pattern changes, it alters the way you think and feel. One shift in the pattern tends to trip another"

-HILARY MANTEL"

Parathyroid hormone (PTH), a signaling molecule produced by the parathyroid gland, is also called parathyrin or a parathormone and keeps the normal regulation of the calcium concentration maintained. It's one of the most vital secretory products. During calcium dysregulation, like in hypocalcemia, it increases calcium's renal absorption and calcitriol's (1,25-Dihydroxycholicalciferol) production, affecting the intestinal calcium absorption, thus helping in the mobilization of calcium from the bones [1]. Along with vitamin D, it also regulates the minerals metabolization in the body, and they together have a feedback cycle [2]. In addition to the functioning of vitamin D, and its regulation, it is also a prime secretory substance playing a part in vitamin D formation [2]. 

Elevated calcium concentrations in hypercalcemia could cause any psychiatric manifestations [3]. There have been several shreds of evidence to date suggesting that high calcium levels in the cerebrospinal fluid (CSF) during hypercalcemia could most likely be a cause of depressive episodes [3]. A case report about a middle-aged woman during a manic episode suggested that despite some maniac or psychiatric symptoms in the past, she had significantly raised PTH levels and calcium [3]. There has also been emerging evidence that voltage-gated calcium channels can depolarize the cell membrane, which may trigger a neurotransmitter release along with synaptic plasticity that, although it is short term, still can cause neuropsychiatric diseases and symptoms [4].

Neurological manifestations could be a result of any alterations in the CSF. For example, the concentration of amyloid-P plaque in Alzheimer's. After years of research, it is identified that any modifications in calcium are significant to Alzheimer's pathophysiology since calcium's misregulation can be directly associated with any alterations of tau phosphorylation and amyloid precursor proteins that define Alzheimer's clinical features [5,6]. A radioimmunological measurement of PTH was done in CSF samples from two patients, one aged 40 years, and the other aged 70 years, with secondary hyperparathyroidism [7]. However, more studies must be conducted to fill the gaps in a higher number of patients with hyperparathyroidism and hypoparathyroidism and do a CSF evaluation further to see any hormone or calcium's abnormal concentrations. More studies with evidence of calcium and magnesium ions played a part in the CSF during ethylic-traumatic coma proven by a simple photometric test. All those with alcoholic-traumatic coma had escalated calcium levels in CSF [8]. This analysis has proven to be very important as a prognostic factor in ethylic-traumatic coma patients [8].

After subarachnoid hemorrhage, calcium's intracellular concentration and regulation in the cerebral vessels alter, and it has been hypothesized that patients have decreased apolipoproteins and high calcium levels may have vasospasm [9]. Moreover, in the treatment of head injury, the CSF is somehow linked. A study analyses CSF's pathophysiology in humans on a molecular and biochemical level showing modifications in any traumatic head injury [10]. 

What is known about the CSF's influence on any neuropsychiatric manifestations due to a dysregulated PTH is the increased calcium concentrations and possible vitamin D concentrations that may affect a patient's neurological health. However, more exploration is required since a few studies have suggested a weak relation of PTH with calcium, but that being the case in studies with fewer patients, without any or significant faulty alteration of PTH in them.

We aim to explore the possibility and the role of PTH dysregulation and, thus, calcium and calcitriol levels to be irregularly distributed, especially in the CSF, to be further causing any symptoms neurological or psychiatric.

In this review article, we will assess the effects of parathyrin on the CSF and how the CSF composition post-PTH-dysregulation could be a possible reason for any symptoms or repercussions relating to the neurological system of a patient's mental health-related quality of life.

## Review

In this review article, we aim to explore the possibility of any linkage between the CSF alterations due to any pathology related to the parathyroid gland, which can, in turn, serve as a cause for neuropsychological repercussions. Neurological and psychiatric symptoms, disorders, and illnesses due to primary hyperparathyroidism have been reported through case reports, case-control studies, cohort studies, and several literature reviews, including the traditional evaluation and systematic.

Physiology and pathophysiology correlating with the clinical aspect

**Figure 1:**
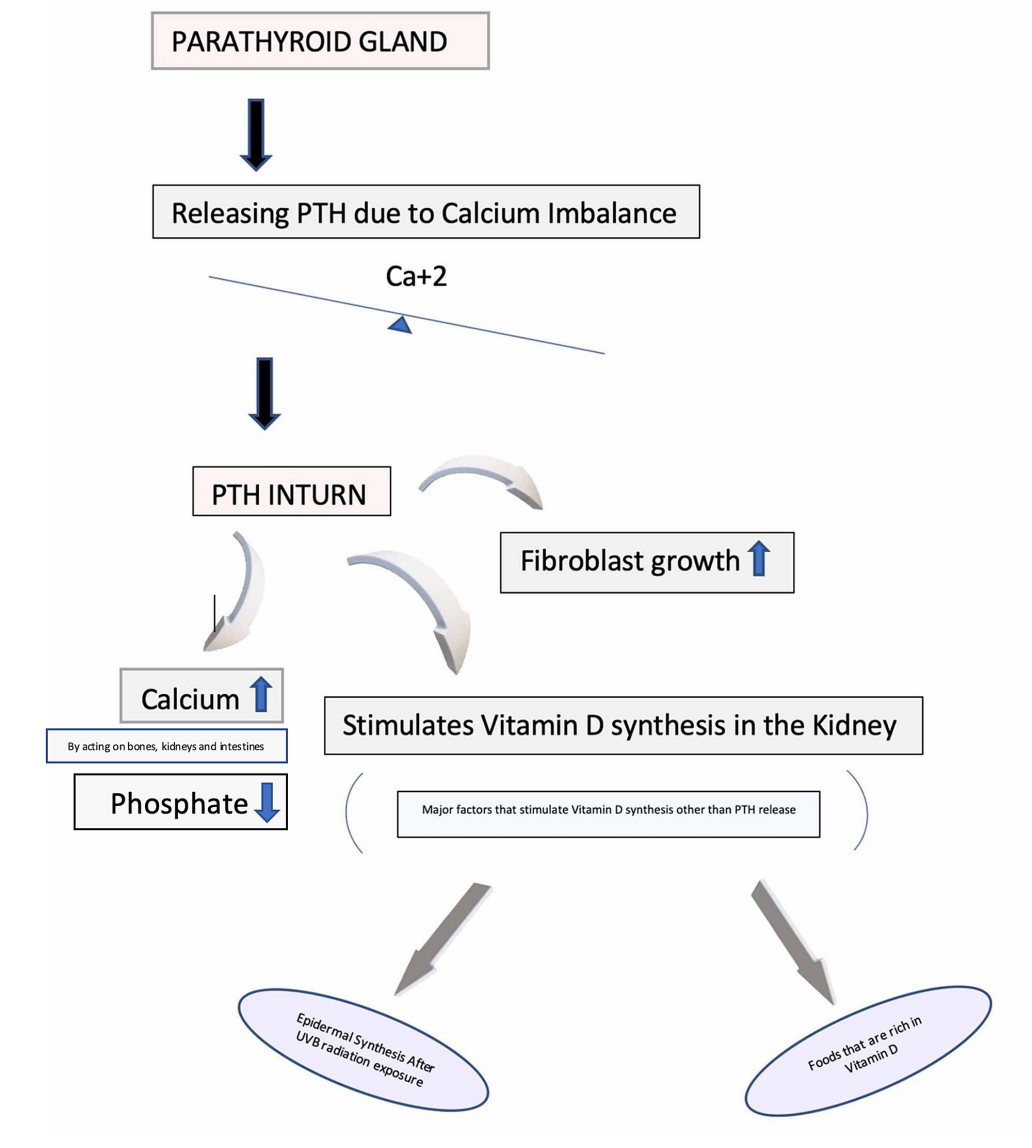
The parathyroid gland due to any sort of calcium imbalance, switches on the negative feedback mechanism, which in turn releases PTH to normalize the concentration of calcium in the body. In addition to increasing the calcium during its deficiency, PTH also decreases phosphate, regulates the growth of fibroblast and, acts on bones, kidneys and, intestines. However, Vitamin D's major source is UVB radiation and dietary sources. PTH: parathyroid hormone; UVB: ultraviolet B radiation.

Due to the imbalance of parathyrin in the body, causing disturbed calcium homeostasis in Primary hyperparathyroidism, there may be not just recurring or infrequent elevations of calcium, but rather consistent [1]. There is a proper feedback loop that regulates the calcium in the blood, which is when the parathormone functions due to its decreased levels [11]. It elevates its absorption from the kidneys and intestines along with releasing it from the bones. Moreover, it also releases fibroblast growth factors, synthesizes vitamin D, and induces other minerals' metabolism, like phosphate in the body [2,11]. Figure 1 explains the preceding sentences explaining the PTH functions and how the negative feedback works.

A few case reports that have been published, one about a 17-year-old adolescent male and one about an 84-year-old woman [12,13], proved the pathophysiology inside that might be hampering or triggering neurophysiological symptoms. The scenario of a 17-year-old suggested psychosis in the patient to be due to significantly elevated calcium and iPTH (intact parathyroid hormone) levels, which were even more strikingly elevated; Around four times that of the upper limit, and the older woman complaining of delusions and psychotic depression coupled with catatonia, who despite lorazepam, was seen to improve very little or not at all [12,13]. However, the patient's detection of persistent hypercalcemia made them diagnose primary hyperparathyroidism and further manage it. The correction of calcium and surgery for removing the parathyroid gland proved significant in alleviating the symptoms [12,13]. Slightly contrary to these, after an unsatisfactory response to olanzapine and then later on gabapentin in a middle-aged woman's psychosis, which was rare, parathyroidectomy too didn't serve a good role, and despite little alleviation in her symptoms in the inpatient psychiatric care, her MMSE (Mini-mental state examination) didn't improve [14]. After being discharged, she was admitted again in later months due to a similar condition [14]. This also proves that even after surgery, a patient would not be relieved of the symptoms and may still need impatient care in the psychiatry ward due to hypercalcemic psychosis [14]. The previously mentioned study can be opposed by another case of a 30-year-old lady who has postpartum psychosis, whose decreased calcium levels upon correction eased her primary hypoparathyroidism [15]. Despite the contradictions, one point remains unchanged: dysregulated calcium in the body serves neuropsychiatric manifestations. The literature review recently published in 2018 strengthens the point even more since its main focus is to help the physicians make an apt diagnosis by taking hypercalcemia as one of the dysregulations linked to psychosis [16]. The review further concluded with the management's suggestion to be seriously considered for high calcium levels in primary hyperparathyroidism [16].

Refractory psychosis, along with excessive consumption of alcohol and seizures, in a 36-year-old man, who had his first convulsion in the first 10 years of his life, was seen with low levels of calcium, high levels of phosphate, and parathyrin levels in the upper limit [17]. With more neurological and psychiatric manifestations like delusions of being persecuted, limb tremors, dysarthria, and CT (computed tomography) brain showing calcifications in the basal ganglia, Fahr's syndrome was diagnosed, which also led to the body failing to respond to the parathyroid hormone (pseudohypoparathyroidism) [17]. Furthermore, to date, evidence specify that hypercalcemia increases the calcium content in the cerebrospinal fluid, thus causing depression. This was backed-up by the case of a maniac female with considerably raised calcium levels due to an elevated parathyroid hormone regulation [3]. Additionally, this reviewed the previously studied facts of how important calcium channel blockers' role is to treat mania [3].

Considering the recency of a study and the number of samples used, which aimed to find out a certain amount of PTHrp (parathyroid hormone-related protein) managing the calcium regulation even in the brain, it holds more significance [18]. Nine diagnostic CSF molecules in 140 paired serums were used. However, the study concluded with PTHrp being labeled as a normal element in the CSF instead of an odd one to cause abnormalities [18]. This does not relate directly to the parathormone, and thus despite the authenticity of this study, there is little to associate it with what we are looking to explore.

To further prove, an article, after being reviewed, implied that calcium's voltage-gated channels if mutated might trigger dysfunction and certain modifications that could serve as a cause of neuropsychiatric diseases and a spectrum that includes ataxia, schizophrenic symptoms, bipolar disorder, migraines, and depressive disorders [4]. Apart from just calcium playing a role due to its voltage-gated channels or short-term synaptic plasticity, more exploration had been done and required to associate any pathophysiology in the human cerebrospinal fluid side by side to determine and co-relate, such as one study proving the presence of elevated calcium in a human CSF during an alcoholic-traumatic coma, and thus this analysis had been concluded as one of the very vital prognostic factors in patients with alcohol induce trauma [8]. Moreover, this study holds more weightage to prove correlating pathophysiology of calcium and CSF since it's reviewed evidently. Another similar analysis was established to evaluate human CSF's pathophysiology but on a more biochemical and molecular level, which showed various alterations during head trauma [10]. A very recent systematic review and meta-analysis of last year and an observational study done two years before explores the possibility of any inflammatory markers in the CSF causing schizoaffective disorder. High levels of inflammatory products are most likely to cause a CSF pathology and the correlation of intracranial injuries with anti-cardiolipin antibodies with c3 [19,20]. In this situation of neuropsychiatric lupus, the most commonly observed symptoms were headaches, seizures, and cerebrovascular accident (CVA) [20]. However, all these points in the above studies need to be further explored to know if calcium and the repercussions of any overactivity of parathyrin can be associated with neurochemical disarrangements and changes in CSF.

Apart from explaining parathyrin's physiology and its clinical importance in vitamin D production and calcium reabsorption from the kidneys, the pathophysiology of parathyroid hormone was also considered, that there is a marked possibility that low levels of vitamin D correlations influence neurological development [1,21]. This was analyzed through an observational study on an animal model [21]. The female rats were kept deprived of vitamin D before they could be bred and also during the dams' birth when given a proper vitamin D diet. Using this observational procedure, it was found that developmental vitamin D deficiency had altered the brain shape with an increased density of the ventricles and alterations in its volume. Moreover, it was also found to be making a difference in the neuronal cells. Thus, the point that it is a convincing factor for any neurological or psychiatric disorder is to be taken into consideration [21]. We believe that this being an animal study isn't strong enough to prove a point in humans, and thus, more investigation should be necessary to establish its effect on human brains.

Case-control studies conducted along with standardized tests for determining the quality of life before and after parathyroidectomy were also favorable. Thirty patients with pHPT (primary hyperparathyroidism) were observed six months before the surgery [22]. Then six months post-surgery to evaluate the difference in psychopathological, neuropsychiatric, and cognitive disabilities after the surgery and thus due to proper observations being carried out during the study and evidence collected, parathyroidectomy was suggested to ease the symptoms in all diagnosed patients even if asymptomatic with slightly impaired cognition [22]. Like this, another case-control research was done to see whether the removal of the parathyroid gland can ameliorate the quality of life by easing the neuropsychological symptoms. The patient evaluated underwent psychometric tests, and a short-form health survey was done post-operatively to know more closely and better their quality of life, health-wise. The severity of the symptoms had dropped down markedly [23]. To prove this strongly, another case-control study that aimed to analyze Calcidiol (25[OHD) levels in patients affecting their mental health demonstrated that the risk of vitamin D deficiency is a possible risk factor in recurrent depression without it associating with the management of depression in any way [24]. This was done by taking 91 patients who met the depression criteria, according to ICD-10 (International Classification of Diseases), and then assessed for any symptoms of depression [24]. The 89 patients of the control group matched according to their age and gender were all healthy [24]. After evaluating, the results showed decreased serum calcidiol levels in those 91 patients with depression [24]. Therefore, again it can be validated how closely related the calcium levels due to parathyrin’s abnormal alterations are to neuropsychiatric health. The various qualities, objectives, types, and conclusions of studies explained in the above passage have been summarized in Table 1 [1-4,8,10-24].

**Table 1:** The various qualities, objectives, types, and conclusions of studies explaining the physiology and pathophysiology in correlation to the clinical aspect. PTH: parathyroid hormone; CSF: cerebrospinal fluid; PTHrp: parathyroid hormone-related protein; CVA: cerebrovascular accident.

Authors	Year of publication	Study type	Objective	Conclusion
Goltzman D [1]	2018	Review Article	Physiology of Parathyrin and its function	Clinically significant for the production of vitamin D, calcium reabsorption from kidneys, and its mobilization in the bones. Besides, it was determined that it may also have a role to play as replacement therapy in hypoparathyroidism.
Khundmiri et al. [2]	2016	Review Article	Correlation of PTH and vitamin D and how they function together to metabolize certain minerals and the feedback mechanism between them	PTH regulates the production of vitamin D also along with regulating calcium in the body and vitamin D may stimulate both phosphate and calcium in the body. Moreover, they can both exert effects on the heart as well.
Brown et al. [3]	2007	Case Report	Roles of elevated levels of parathyrin and calcium, causing episodes of mania	High calcium levels to possibly cause psychiatric symptoms including mania
Nanou and Catterall [4]	2018	Review Article	Physiology of voltage-gated calcium channels and their part in causing synaptic plasticity that in turn may manifest and psychiatric or neurological consequences.	Triggering of neurotransmission due to the influx of calcium can be altered due to gene mutations and polymorphism of calcium channels. This was seen to cause short-lived synaptic plasticity that proved to be potent enough to cause any neuropsychiatric illness
Bologa et al. [8]	2003	Review Article	Analyzing the role of magnesium and calcium in the CSF during traumatic coma because of alcoholic overdose	Patients with traumatic coma due to high intake of alcohol showed the presence of high levels of calcium and a decrease in magnesium level post photometric test
Bakay et al. [10]	1986	Review Article	Role of CSF in molecular and biochemical ways during head trauma	Importance of CSF in prognosis, diagnoses, and management of any pathophysiological symptoms during and after head injury
Goltzman et al. [11]	2018	Review Article	Physiology of how Calcium and vitamin due to parathyroid hormone function in the body and vice versa	Renal and intestinal reabsorption of calcium along with its decreased release due to low PTH levels, Calcium sensing receptor stimulation, and suppressed synthesis of 1,25(OH)2D.
Babar and Alemzadeh [12]	2014	Case Report	Pathophysiology of parathyroid gland causing calcium disruption. Thus, presenting psychosis in young male	Frank psychosis mainly manifested in an adolescent and not in pediatric, middle-aged of old age population due to Hypercalcemia
Parks et al. [13]	2017	Case Report & Review	Studying the relation of psychopathology with increased levels of calcium during hyperparathyroidism after a case of an aged woman with mild hypercalcemia and a continuously elevated parathyrin manifesting psychotic symptoms, later diagnosed with primary hyperparathyroidism	The evidence indicated that neuro-psychosis may improve in PHPT post-parathyroidectomy.
Nagy et al. [14]	2020	Case Report	Studying the link between a middle-aged woman’s psychosis and her elevated calcium levels, a rare hospital course, and the lady’s management	After an unsatisfactory response to olanzapine, and then gabapentin, and a few more proposals to its management, surgery was still recommended, and in some cases inpatient psychiatric care.
Patil et al. [15]	2010	Case Report	Study on a 30-year-old female suffering from postpartum psychosis to evaluate the possible relation of parathyroid disorder	The lady had a notable decrease in calcium levels during this period and upon calcium correction, she improved, thus being diagnosed as a patient of primary hypoparathyroidism
Bojdani et al. [16]	2018	Case Report & Review	To help the physician in making an apt diagnosis by taking hypercalcemia as one of the dysregulations linked to psychosis as one of its causes	Suggestions For the management of elevated calcium levels thus causing psychosis in a patient with primary hyperparathyroidism
Otheman et al. [17]	2011	Case Report	To explore the correlation between neuro-psychosis coupled with seizures and Fahr’s syndrome and if there’s possible pseudohypoparathyroidism	Fahr’s syndrome to be considered as one of the differential diagnosis if evaluating a neuro-psychotic patient with convulsions and also consider neuroimaging to look for any interferences during phospho-calcic metabolism
Kushnir et al. [18]	2018	Observational	To find out if a certain amount of PTHrp is found in the human CSF and serum	PTHrP normally present in the human CSF in a significantly larger amount than in the serum and directly proportional to age and elevated in women as compared to men
Orlovska-Waast et al. [19]	2019	Systematic Review and Meta-analysis	Exploring the possibility of the presence of inflammatory markers in CSF causing schizoaffective disorders	Patients with schizoaffective disorder can most likely have a CSF pathology with elevated inflammatory products and markers, immunoglobulins, and also autoantibodies
Tan et al. [20]	2018	Observational study`	To survey and scrutinize the clinical signs and symptoms, laboratory assessment, imaging, and role of autoantibodies in characteristics of neuropsychiatric lupus to reach a proper outcome	The most commonly observed and stated manifestations were headaches, seizures, and CVA. The CSF testing and MRI were seen to be playing a vital role in its assessment and a correlation of intracranial injuries with anti-cardiolipin antibodies along with c3 was seen
Eyles et al. [21]	2009	Observation Study	Association of the deficient of Vit D and the maturing of Brain studies in rats	Vitamin D could pose a risk biologically for any neuropsychiatric illness considering it may function as a neuro-steroid that can affect the neurological maturing
Dotzenrath et al. [22]	2006	Case-Control	To observe the patients by following up post-parathyroidectomy, to see any cognitive and other mental improvements to prove a long-term recovery	Improvement in cognition six months after surgery for primary Hyperparathyroidism
Weber et al. [23]	2007	Case-Control	How parathyroidectomy can affect the quality of life due to any neurological or psychiatric repercussion in primary hyperparathyroidism	Patients 12 months post-surgery for follow-up showed diminished and depressive symptoms and presented with little or no anxiety
Józefowicz et al. [24]	2014	Case-control study	To analyze 25[OH]D concentration in victims with ongoing depression and if its symptoms and intensity depends on calcidiol	The risk of being vitamin D deficient was concluded as a possible risk factor in recurrent depression without its association with the treatment of depression

Cellular and molecular associations

On a molecular and a cellular level, we are exploring the causative elements, mechanisms, and the changes taking place that hold significance in understanding how the parathyroid gland, when dysfunctional, can affect the hormonal irregularities and thus put an impact on neurological and psychiatric health, by altering the CSF. A case-control study done in 2011 focused on assessing the concentration of magnesium, calcium, and other minerals like copper, zinc, manganese, and iron in the CSF [25]. one-seventy-three samples of CSF that were looked at showed 136 to be the ones with neurological pathology [25]. After a detailed evaluation, the results showed a significant rise in calcium concentration and some other elements in the CSF of the pathological group [25]. Another study done with a control group suggested a contradicting result, in which the pathological group with an endocrine disorder (hyperparathyroidism) showed no significant rise of calcium in the CSF as compared to the plasma, and there was a weak link of calcium with PTH in the CSF [6]. Despite it having specified the age groups and proper evaluation, the article's age and a smaller number of samples make it weigh less than the study mentioned before, so more probing is needed. A cross-sectional study mainly focusing on acetylcholinesterase's functioning along with 25 hydroxyvitamin D levels changing in those with Alzheimer's in the CSF showed decreased levels of 25OHD and Acetylcholinesterase activity but without little or no association of them both in the CSF [26]. Comparatively, to discuss more associations, on a biochemical and cellular level, an observational study shows the link of apolipoprotein E with calcium levels after SAH (subarachnoid hemorrhage) in the CSF, without any influence on the vasospasms [9]. However, in another study that was done to inspect the link between IgG's intrathecal synthesis and vitamin D levels in the CSF of patients of multiple sclerosis, no significant influence of vitamin D on the IgG synthesis was seen despite assuming a probability of vitamin D altering B-cell function that can lessen the synthesis. [27]. To build up the point, a recent article published three years ago by a calcinist discussed the activity of calcium in AD (Alzheimer's disease) and interactions of mitochondria [6]. It suggested that altered tau phosphorylation, the amyloid precursor protein's processing, and synaptic abnormality can be related to calcium dysregulation [6]. Any amyloidopathy can trigger any sleep dysregulation and make the brain more unguarded, increasing the chance of Alzheimer's [5,6]. A better inquiry is needed to use calcium as a therapeutic agent in Alzheimer's. What more can make the brain vulnerable and hinder neurological development is Vitamin D, according to a literature review. This also links vitamin D's insufficiency to schizophrenia; however, no definite evidence was found to accept vitamin D's role in them [28]. SPA (serum prolidase activity) levels surged in choroid plexus calcification, and a remarkable link between SPA and vitamin D levels is seen along with a slight correlation to Parathyroid hormone as well but not significantly [29]. Prolidase is an enzyme for breaking the protein. It is shown through the above study that it has a link with vitamin D levels, and since parathyrin's activity can regulate vitamin D concentration in the serum, is there a possibility that it may affect serum prolidase, which is said to be extremely elevated during choroid plexus calcification? The point that vitamin D and choroid plexus could be linked should be considered, but more observational studies are needed.

According to cellularity, a review article of 2017 indicated the connection between vitamin D and depression through the discussion [30]. Changes in neural mechanisms and triggering of the neurons are connected to intracellular calcium levels, and its role also proved why deficient levels of vitamin D could pose a risk for psychoneurological diseases like depression [30]. The various qualities, objectives, types, and conclusions of studies explained in the above passage have been summarized in Table 2 [5-7,9,25-30].

**Table 2:** The various qualities, objectives, types, and conclusions of studies relating to the cellular and molecular aspect of the topic. CSF: cerebrospinal fluid; AD: Alzheimer’s disease; PTH: parathyroid hormone; SAH: subarachnoid hemorrhage; SPA: serum prolidase activity.

Authors	Year of publication	Study type	Objective	Conclusion
Boespflug and Iliff [5]	2018	Review Article	To establish if there is an association between the CSF and Interstitial fluid exchange, sleep and a hallmark of AD that is amyloid-β	Outlines the fact that age-related sleep hampering can be a factor in serving amyloidopathy and makes the brain more expose and increased the chances of AD as well. Also, amyloidopathy on the cellular level may cause sleep disturbances.
Gibson and Thakkar [6]	2017	Review Article	Associating The activity of calcium in AD (Alzheimer’s disease) and Interactions of Mitochondria and its mechanisms	Altered tau phosphorylation, processing of the amyloid precursor protein, and synaptic abnormality can be related to calcium dysregulation, however, more understanding is needed on this.
Balabanov et al. [7]	1984	Case-Control study	Measuring the PTH radio immunologically and finding out its relation in the CSF with magnesium and calcium	The study proved that PTH is normally present in the CSF and there is no association of Magnesium and PTH in the human CSF and weak link between CSF’s calcium levels
Alexander et al. [9]	2008	Case-Control study	To hypothesize if there is any association of Apolipoprotein E in CSF, calcium, and possibility of cerebral vasospasm after SAH	Correlation of Apolipoprotein E with calcium levels in the CSF after SAH, without influencing any vasospasms.
Romaris et al. [25]	2011	Case-Control study	To inspect the presence and concentration of calcium, magnesium, and other trace elements in the CSF	Elements Ca, Cu, Fe, Zn, and Mn in pathological groups were elevated in the CSF as compared to those which were healthy.
Johansson et al. [26]	2013	Cross-Sectional study	If the acetylcholinesterase functioning along with 25 hydroxyvitamin D levels changes in patients with Alzheimer’s in the CSF	Decreased levels of 25OHD and Acetylcholinesterase activity in the CSF in Alzheimer’s disease but little or no association between AChE activity and 25OHD.
Holmøy et al. [27]	2012	Observational Study	To inspect any link between the IgG’s intrathecal synthesis and levels of Vitamin D in the serum and CSF in MS (multiple sclerosis)	No significant influence of the physiological changes in Vitamin D on the IgG Intrathecal synthesis in MS
Amaral et al. [28]	2014	Review Article	To find out If insufficiency of vitamin D can hinder neurological development and is associated with schizophrenia	No strong and definite evidence to accept vitamin D’s role in schizophrenia and the pathways and anatomy that is disrupted neurologically due to vitamin D’s insufficiency in schizophrenia.
Kaleli et al. [29]	2016	Case-Control study	The action of SPA (Serum Prolidase) as a biomarker for the CPC (choroid plexus calcification)	SPA level was notably surged in the CPC group and a marked link between SPA and Vitamin D levels, along with it linking to PTH levels as well but not very significantly.
Berridge [30]	2017	Review Article	The connection between Depression and vitamin D considering the cellularity and regulation in the body	Changes in the neural mechanism and triggering of the neurons are connected to the intracellular levels of Calcium and its role also proves why deficient levels of vitamin D can pose risk for psychoneurological diseases like depression.

Psychoneuroendocrinological correlations

**Figure 2:**
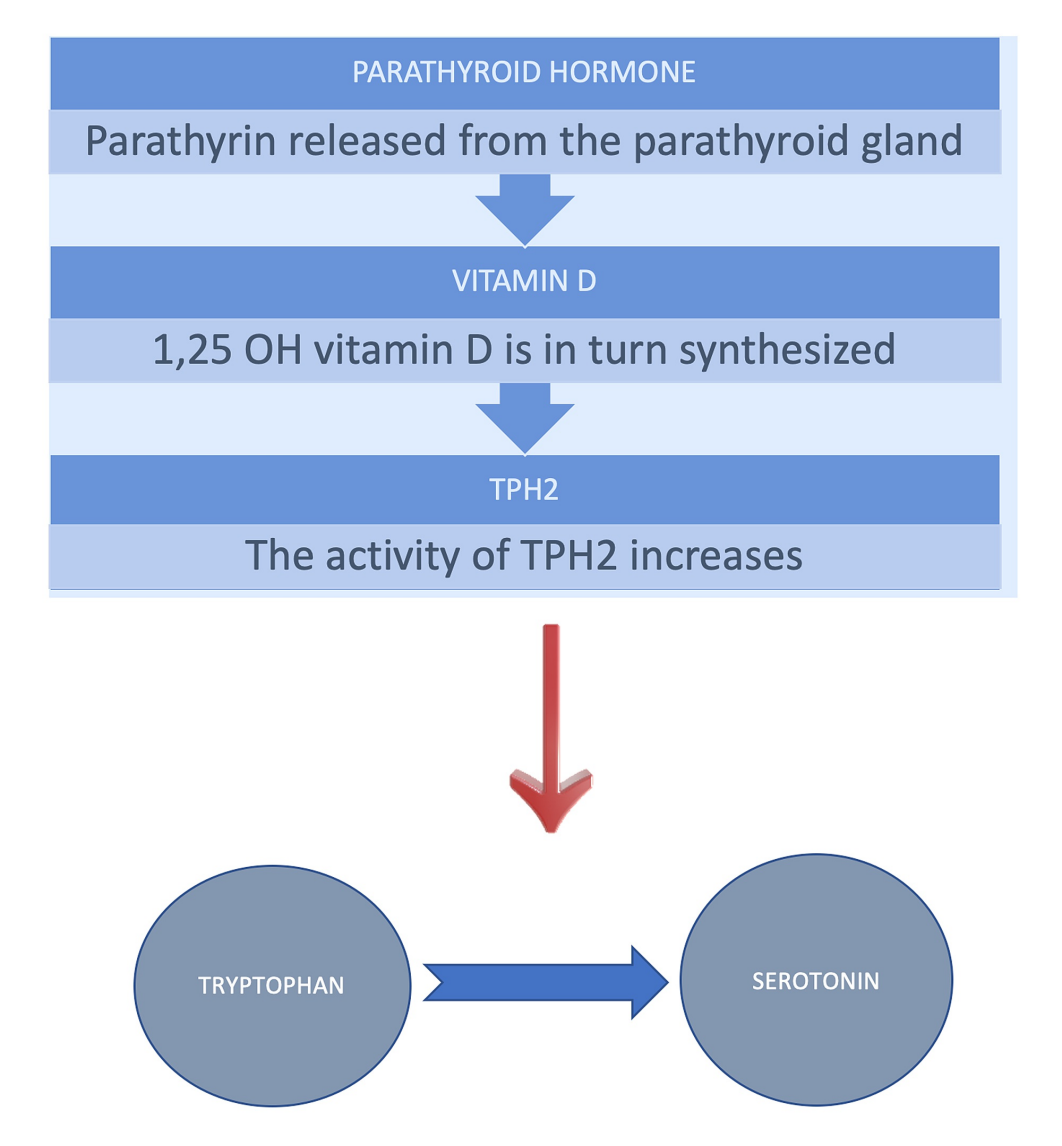
The release of parathormone from the parathyroid gland, synthesizes vitamin D, which in turn produces TPH2. This enzyme is responsible for the conversion of tryptophan into serotonin.

**Figure 3:**
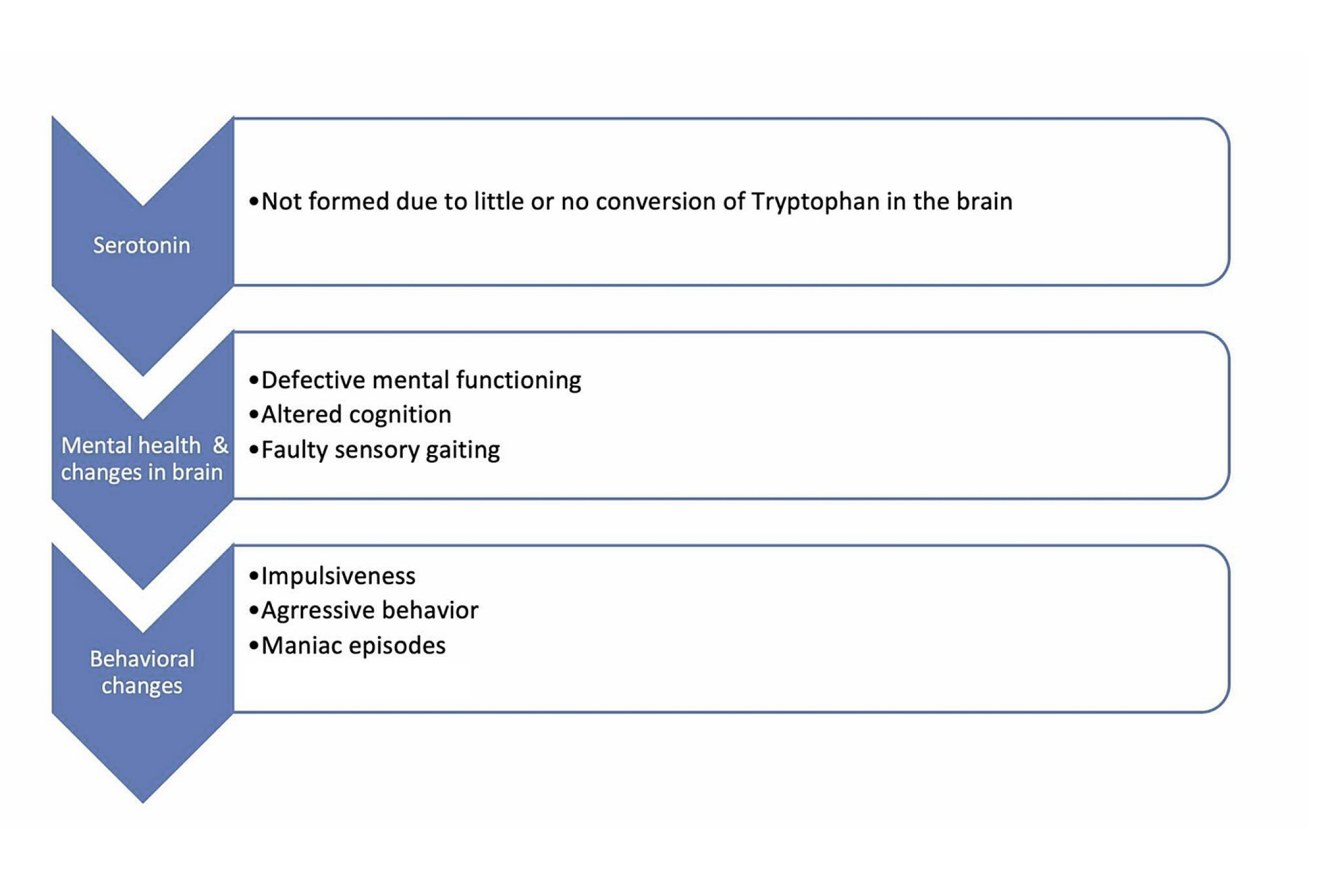
Due to little or no serotonin formed, because of the cessation of tryptophan's conversion in the brain, an affected individual's mental health starts compromising. Along with a defective mental state, there is altered cognition and faulty sensory gaiting. Furthermore, in terms of behavior, the patient experiences impulsiveness, outbursts, aggression, and manic episodes.

To associate psychoneurological manifestation and mechanisms with those of the endocrine system is vital to emphasize and investigate their associations in the human body. Adamson et al. explore the possibility of how psychotic symptoms and illnesses can be related to low vitamin D levels [31]. This systematic study included 53 new associations of vitamin D deficiency repercussions in those with psychosis [31]. Still, despite all the studies collected, the basis was weak, considering the ambiguity of the evidence's shreds and their inconsistent associations [31]. However, the authors in a literature review showed strong empirical pieces of evidence from previous works of literature about the association of low levels of vitamin D in depressed patients and if any management including vitamin D can be of any importance in these patients, but again more empirical studies are needed for more clarity [32]. Another literature review gave strong evidence of how vitamin D levels can affect serotonin production in the brain by affecting the tryptophan's movement into the blood-brain barrier [33]. Suppose ample supplementation including vitamin D and omega-3 fatty acids, is given. In that case, it can improve the behavioral changes seen in serotonin deficient patients by normalizing the serotonin levels, who may have impulsive and aggressive behavior along with some other mental illnesses [33]. Figure 2 and Figure 3 summarize the mechanism mentioned above through illustrations.

Similarly, an observational study from last year discussed the role of vitamin D and depression properly. It showed that vitamin D insufficiency was remarkably and often seen in those with acute stroke, and its low levels link to depression being caused in acute stroke [34]. This was concluded by observing 442 patients over weeks [34]. Another literature reviewed the associations and proposed the changes in neural mechanism and triggering of the neurons connected to intracellular calcium levels and how glutamate could be one reason [30]. The study was concluded with clarity that the part vitamin D play intensifies surging calcium in maintaining respiration in mitochondria and how it regulated the serotonin synthesis [30]. They also concluded by saying that it maintains the neurons' normal activity to prevent depression [30]. Contrary to this, another literature after reviewing studies concluded that no definite and robust evidence is seen to accept vitamin D's role in one of the psychiatric disorders, like Schizophrenia, and the pathways along with anatomy are disrupted in the neurological system due to vitamin D insufficiency [28].

A case report in 2016 discussed an aged man with organic psychosis due to deficient functioning of the parathyroid gland due to any therapeutic or diagnostic procedures (Iatrogenic) [35]. The report exemplified that a workup must be seen for psychosis if patients with psychiatric issues present atypically [35]. The symptoms can be hypoparathyroidism, low calcium levels, and brain calcifications [35]. A case-control study quantified the concentrations of 25OHD3 in the CSF and serum of the patients with relapses of multiple sclerosis and observed no significant influence of 25OHD3 on CSF concentration [36]. However, a slight decrease in those with multiple sclerosis was seen [36]. Considering it's a case-control study, it holds more weightage and stronger evidence, and thus the fact that parathyrin can regulate vitamin D and calcium and further vitamin D in any form can affect one's neurology, can't be denied entirely, and considering the study type, it can be given more leverage than the previously mentioned case.

To sum it all up, there aren't many studies discussing a direct linkage between parathormone causing CSF pathology so that it directly and as the only pathology that affects a human's neurological or psychiatric state. Despite many studies proving how calcium and vitamin D levels, along with their other forms, can cause neuropsychological manifestations, we are still unsure if their altered levels in the body are solely dependent on the parathyroid gland's functioning.

Is there a possibility of Parathyroid hormone after entering the CSF to change its form in a way other than just dysregulating calcium that causes a direct pathology and abnormal modifications in the brain? That is yet to be explained and proved by empirical studies collecting huge and high CSF samples. The various qualities, objectives, types, and conclusions of studies explained in the above passage have been summarized (Table 3) [28,30-36].

 

**Table 3:** The various qualities, objectives, types, and conclusions of studies relating to the psychoneuroendocrinological associations.

Authors	Year of publication	Study type	Objective	Conclusion
Amaral et al. [28]	2014	Review Article	To find out If insufficiency of vitamin D can hinder neurological development and is associated with schizophrenia	No strong and definite evidence to accept vitamin D’s role in schizophrenia and the pathways and anatomy that is disrupted neurologically due to vitamin D’s insufficiency in schizophrenia
Berridge [30]	2017	Review Article	The connection between depression and vitamin D considering the cellularity and regulation in the body	Changes in the neural mechanism and triggering of the neurons are connected to the intracellular levels of calcium and its role also proves why deficient levels of vitamin D can pose risk for psychoneurological diseases like depression.
Adamson et al. [31]	2017	Systematic Review	Evidently, look into the linkage between psychosis and vitamin D levels	Indefinite evidence to prove the association of psychosis and vitamin D and thus more studies are required
Parker et al. [32]	2017	Review Article	If deficient vitamin D has any association with depression and depressive symptoms or if putting someone on vitamin D medication can affect this illness	Empirical studies suggest a strong link between the two, however, studies are needed for the clarity of its effect on depression and whether or not it should be considered as one of the managements.
Patrick and Ames [33]	2015	Review Article	To check if vitamin D can control the production of serotonin and thus control the symptoms in serotonin deficient patients	The increment of vitamin D in the body and also omega-3 fatty acid through supplements can elevate the serotonin in the brain and thus restore normal behavior in an individual and limits aggression and impulsiveness
Gu et al. [34]	2019	Observational study	To determine if vitamin D alters according to season and if it causes depression in those with acute stroke	Insufficient vitamin D was markedly and very frequently seen in those with acute stroke and its low levels link to it causing depression in acute stroke
Amaral et al. [35]	2016	Case Report	Discussing a case of an aged man with organic psychosis due to deficient functioning of the parathyroid gland due to any therapeutic or diagnostic procedures (Iatrogenic)	The report exemplified that a workup is required to see for organic psychosis if patients with psychiatric issues come with atypical symptoms and also that the symptoms can be due to hypoparathyroidism, low levels of calcium, and brain calcifications.
Moghtaderi et al. [36]	2013	Case-Control study	To quantify the concentrations of 25-OH-D3 in serum and cerebrospinal fluid of the patients presenting with relapsing multiple sclerosis	No major influence of 25-OH-D3 was found on the cerebrospinal fluid concentration; however, there was a slight decrease in those with MS comparatively.

Limitations

Randomized controlled trials were not included in the article, and we had only gathered observational, literature, and case studies. There were not many studies that proved a direct link between the parathyroid gland and its dysfunctional parathormone regulation, which in turn can make any direct changes inside the CSF that can cause pathology or lead to any neuropathological disease solely. Additionally, not many studies included were diverse, considering we only gathered mostly those in English. There could have been a possibility of articles directly linking the two in any other language. Moreover, we mainly stuck to articles mostly from the last 20 years except one, and thus had we explored more articles despite their recency, more information could have been extracted.

## Conclusions

Our review pointed towards the parathormone's concentration and its regulation by the parathyroid gland that can disrupt the neurological and psychiatric system and function by causing any alterations in the CSF. The review was vital to clarify and give enough proofs to establish that markedly elevated and decreased calcium levels both in the serum and CSF can be responsible for affecting one's neuropsychological health. This is to be considered since calcium dysregulation of any sort due to abnormal parathormone flow can cause manifestations and illnesses like cognitive disability, delusions, schizophrenia, schizoaffective disorder, Alzheimer's disease, convulsive episodes, coma in rare cases, and most commonly, depression and other psychotic disorders, however, in some cases, it may be reversible and some mental chronic illnesses may have normal calcium levels. Moreover, it's not just calcium but also how vitamin D insufficiency can lead to depression and hamper neurological well-being. It also provided a few observational studies and analyses that are substantial to show how even parathyroidectomy in hyperparathyroidism cases can serve in improving the quality of life. However, the hypothesis that CSF changes due to parathormone's regulatory dysfunction can play a part in neuropsychological manifestations by altering the calcium or vitamin D concentrations in the body couldn't be authenticated considering we didn't find or include enough substantial studies to validate it, except a few showing increased calcium in CSF as one of the causes for neuropsychiatric features and some other pathologies in the nervous system excluding the CSF. Thus, there is a need to study this more and perform observational studies that are done for a more extended period and include many patients.
